# A plain language summary of the long-term relugolix combination therapy study for uterine fibroids

**DOI:** 10.57264/cer-2023-0069

**Published:** 2023-07-21

**Authors:** Ayman Al-Hendy, Andrea S Lukes, Roberta Venturella, Claudio Villarroel, Laura McKain, Yulan Li, Rachel B Wagman, Elizabeth A Stewart

**Affiliations:** 1 University of Chicago, Chicago, IL, USA; 2 Carolina Woman's Wellness Center, Durham, NC, USA; 3 University of Magna Graecia, Catanzaro, Italy; 4 Instituto de Investigaciones Materno Infantil (IDIMI), University of Chile, Santiago, Chile; 5 McKain Consulting, LLC, Las Vegas, NV, USA; 6 Myovant Sciences Inc, Brisbane, CA, USA; 7 Mayo Clinic & Mayo Clinic Alix School of Medicine, Rochester, MN, USA

**Keywords:** anemia, lay summary, menstrual bleeding, menstrual blood loss, plain language summary, relugolix, relugolix combination therapy, uterine fibroids

## Abstract

**What is this summary about?:**

This is a summary of a research study (known as a clinical trial) called the LIBERTY extension study. The LIBERTY extension study is a long-term study looking at how well a medicine called relugolix combination therapy worked in reducing blood loss during menstrual periods in women with uterine fibroids with heavy menstrual periods. Women were included in the extension study if they finished the 24-week LIBERTY 1 or LIBERTY 2 studies. Heavy menstrual periods were considered to be menstrual blood loss of about one-third of a cup of blood (80 ml) per cycle for two cycles or about two-thirds of a cup of blood (160 ml) during one cycle. The LIBERTY extension study also looked at whether relugolix combination therapy was safe to take for up to 1 year.

**What were the results?:**

Out of 770 total women with uterine fibroids with heavy menstrual bleeding who took part in the LIBERTY 1 and LIBERTY 2 studies, 476 took part in the LIBERTY extension study. From the start of the LIBERTY 1 and LIBERTY 2 studies through the end of the LIBERTY extension:

- 163 women took relugolix combination therapy for 52 weeks
- 149 women took relugolix alone for 12 weeks followed by relugolix combination therapy for 40 weeks
- 164 women took placebo for 24 weeks followed by relugolix combination therapy for 28 weeks

The LIBERTY extension study showed that most women in all three treatment groups responded to relugolix combination therapy by having less bleeding during their menstrual periods, having improved anemia symptoms, and having stable bone mineral loss. Side effects were similar across treatment groups, and the most common side effects were headaches and hot flushes.

**What do the results mean?:**

Women with uterine fibroids with heavy menstrual bleeding taking relugolix combination therapy may have fewer uterine fibroid bleeding symptoms for up to 1 year of treatment.

**Clinical Trial Registration:** NCT03049735 (ClinicalTrials.gov) (LIBERTY 1)

**Clinical Trial Registration:** NCT03103087 (ClinicalTrials.gov) (LIBERTY 2)

**Clinical Trial Registration:** NCT03412890 (ClinicalTrials.gov) (LIBERTY extension study)

**This is an abstract of the Plain Language Summary of Publication article.**

To read the full Plain Language Summary of this article, click here to view the PDF.

Link to original article here

